# Development of a porcine model of skin and soft‐tissue infection caused by *Staphylococcus aureus*, including methicillin‐resistant strains suitable for testing topical antimicrobial agents

**DOI:** 10.1002/ame2.12495

**Published:** 2024-10-31

**Authors:** Filip Raška, Břetislav Lipový, Šárka Kobzová, Lukáš Vacek, Rea Jarošová, Dominika Kleknerová, Katarína Matiašková, Peter Makovický, Monika Vícenová, Edita Jeklová, Roman Pantůček, Martin Faldyna, Lubomír Janda

**Affiliations:** ^1^ Department of Burns and Plastic Surgery, Faculty of Medicine Institution Shared with University Hospital Brno, Masaryk University Brno Czech Republic; ^2^ Department of Burns Medicine, Third Faculty of Medicine Charles University and University Hospital Kralovske Vinohrady Vinohrady Prague Czech Republic; ^3^ CEITEC—Central European Institute of Technology, Brno University of Technology Brno Czech Republic; ^4^ Department of Infectious Diseases and Preventive Medicine Veterinary Research Institute Brno Czech Republic; ^5^ Department of Biochemistry, Faculty of Science Masaryk University Brno Czech Republic; ^6^ Department of Microbiology St. Anne's University Hospital Brno and Faculty of Medicine, Masaryk University Brno Czech Republic; ^7^ Department of Morphology, Physiology and Animal Genetics, Faculty of AgriSciences Mendel University in Brno Brno Czech Republic; ^8^ Department of Histology and Embryology, Faculty of Medicine University of Ostrava Ostrava Czech Republic; ^9^ Department of Experimental Biology, Faculty of Science Masaryk University Brno Czech Republic

**Keywords:** animal model, antimicrobial agents, porcine model, skin and soft‐tissue infection (SSTI), *Staphylococcus aureus*, wound infection

## Abstract

**Background:**

In view of the ever‐increasing representation of Staphylococcus spp. strains resistant to various antibiotics, the development of in vivo models for evaluation of novel antimicrobials is of utmost importance.

**Methods:**

In this article, we describe the development of a fully immunocompetent porcine model of extensive skin and soft tissue damage suitable for testing topical antimicrobial agents that matches the real clinical situation. The model was developed in three consecutive stages with protocols for each stage amended based on the results of the previous one.

**Results:**

In the final model, 10 excisions of the skin and underlying soft tissue were created in each pig under general anesthesia, with additional incisions to the fascia performed at the base of the defects and immediately inoculated with Staphylococcus aureus suspension. One pig was not inoculated and used as the negative control. Subsequently, the bandages were changed on Days 4, 8, 11, and 15. At these time points, a filter paper imprint technique (FPIT) was made from each wound for semi‐quantitative microbiological evaluation. Tissue samples from the base of the wound together with the adjacent intact tissue of three randomly selected defects of each pig were taken for microbiological, histopathological, and molecular‐biological examination. The infection with the inoculated S. aureus strains was sufficient during the whole experiment as confirmed by both FPIT and from tissue samples. The dynamics of the inflammatory markers and clinical signs of infection are also described.

**Conclusions:**

A successfully developed porcine model is suitable for in vivo testing of novel short‐acting topical antimicrobial agents.

## Graphical abstract

In our paper, we describe several stages of a porcine model of deep skin and soft‐tissue infection caused by *S. aureus*, including methicillin‐resistant strain development. For a successful introduction and easily reproducible immunocompetent porcine model with monoculture infection, we had to pass several consecutive stages with significant modifications. Finally in stage III, after the implementation of all changes, we were able to successfully induce and maintain deep skin and soft‐tissue infection, which was also confirmed by microbiological, histopathological, molecular‐biological, and clinical evaluation.

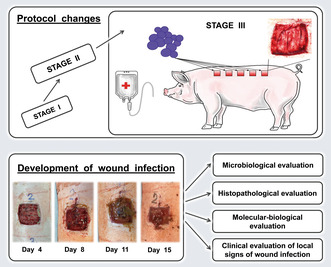

## 1 INTRODUCTION

Infectious complications in patients with acute or chronic wounds count among the most pressing issues, leading to increased morbidity, mortality, and health care–associated costs.^1^ In patients with acute injuries or with early chronic wounds, the intact skin cover, one of the most significant barriers against infections, is completely compromised.^2^ Skin and soft‐tissue infections (SSTIs) represent the most common type of complication in these patients.^3^

Staphylococci, especially *Staphylococcus aureus*, are the most common cause of SSTIs.^4^ This microorganism can acquire several antimicrobial resistance mechanisms, with methicillin‐resistant strains (methicillin‐resistant *S. aureus* [MRSA]) being the best known among these.^5^, ^6^ The use of vancomycin for the treatment of infections caused by MRSA also leads to reduced sensitivity of *S. aureus* to this glycopeptide antibiotic.^7^

*In vivo* efficacy assessment of new cutaneous antimicrobial substances is a necessary phase in the evaluation of novel drugs. Creating an animal model of SSTIs caused by a given pathogen is, therefore, a crucial step.^8^ The pig is considered the ideal model for studying human skin because of its anatomical and physiological similarities to humans. The epidermis and dermis of the pig are thick, with the epidermis ranging from 30 to 140 μm.^9^, ^10^ Pigs as well as humans show developed rete ridges and pars papillaris, and have subdermal fatty tissue. Unlike the skin of rodents, rabbits, and canines, which is loose and slides over the subcutaneous fascia, porcine skin is adherent to the underlying structures.^11^ Neither pigs nor humans have an extensive panniculus carnosus, which is found in small loose‐skinned animals, such as the mouse and rat.^12^ Similarities between pigs and humans are evident also in adnexal structures, although minor differences can be observed. Eccrine sweat glands are restricted to the snout, lips, and carpal organs. Humans, on the contrary, have eccrine sweat glands distributed over the entire body.^13^

In this study, we aimed to develop an infection model of extensive porcine skin and soft‐tissue damage suitable for evaluating new antimicrobial agents against *S. aureu*s strains, including methicillin‐resistant strains, in fully immunocompetent pigs.

## 2 MATERIALS AND METHODS

### 2.1 Bacterial strain selection and characteristics

Based on the multilocus sequence typing (MLST) of human and veterinary *S. aureus* strains, 16 candidate sequence type (ST) strains were selected to obtain the most heterogeneous representatives of each clonal cluster. They have been tested for susceptibility to antimicrobial agents in vitro. Based on the comparison of the growth curves of individual strains, eight candidate sequence types (ST7, ST22, ST30, ST80, ST88, ST225, ST395, and ST398) were selected and subsequently used to induce infection of pigs in stage I of the experiment (see Table 1).^14^ In the next stages, we always applied three of them: ST22 (NRL/Atb 5921), ST30 (NRL/St 08/192), ST395 (PS187). The strains had exhibited very different susceptibility to enzybiotics, biofilm formation capabilities, and resistance to methicillin. ^15^, ^16^

**TABLE 1:** List of eight different sequence types (ST7, ST22, ST30, ST80, ST88, ST225, ST395, and ST398), strain number, genotype, origin, year of strain acquisition, source, clinical diagnosis, and other characteristics.

Strain	Genotype (sequence type/staphylococcal cassette chromosome type/*spa*‐type)	Origin	Year of isolation	Source	Clinical diagnosis	Material	Other characteristics
A23070	ST7/SCC*mec*V/t091	Human	2012	St. Anne's University Hospital Brno (Czech Republic)	Atherosclerosis heart disease	Throat swab	–
NRL/Atb 5921	ST22/SCC*mec*IV/t020	Human	2005	Hradec Králové University Hospital (Czech Republic)	Chronic kidney disease	Bloodstream	–
NRL/St 08/192	ST30/SCC*mec*IV/t019	Human	2008	Motol University Hospital (Czech Republic)	Skin abscess	Purulent discharge	Pannton‐Valentine leukocidin‐positive
NRL/Atb 1615	ST80/SCC*mec*IV/t044	Human	2008	Nathional Institute of Public Health, Praha (Czech Republic)	General medical examination	Nasal swab	–
NRL/St 04/754	ST88/SCC*mec*IV/t186	Human	2004	Nathional Institute of Public Health, Praha (Czech Republic)	Gluteal abscess	Purulent discharge	Pannton‐Valentine leukocidin‐positive
UL SA1	ST225/SCC*mec*II/t003	Human	2008	Regional Health Corporation – Masaryk Hospital in Ústí nad Laben (Czech Republic)	Purulent conjunctivitis	Purulent discharge	–
PS187	ST395/non‐SCC/t8605	Human	1955	UK, culture collection (NCTC 9754)	Case of septicemia	Bloodstream	Propagation strain for phage 187
UMCG‐B	ST398/SCC*mec*V/t034	Human	2003	University Medical Center Groningen (The Netherlands)	Pneumonia	Pleural fluid	–

### 2.2 Inoculum preparation

Bacterial strains stored at −80°C were inoculated into Tryptic Soy Broth (TSB) medium the day before the experiments and incubated overnight at 37°C. In the morning, bacterial strains were subcultured in fresh medium for 4 h to reach mid‐exponential growth phase. Bacterial inoculum was prepared by repeated centrifugation and washing of bacterial suspensions with phosphate‐buffered saline (pH = 7.4). Final bacterial inoculum concentrations were 1.8 × 10^9^ and 6.6 × 10^10^ colony‐forming units per milliliter (CFU/mL) for the first and second phases of the experiment and 2.0 × 10^9^ CFU/mL for the third phase.

### 2.3 Development of wound infection and wound management

**FIGURE 1:**
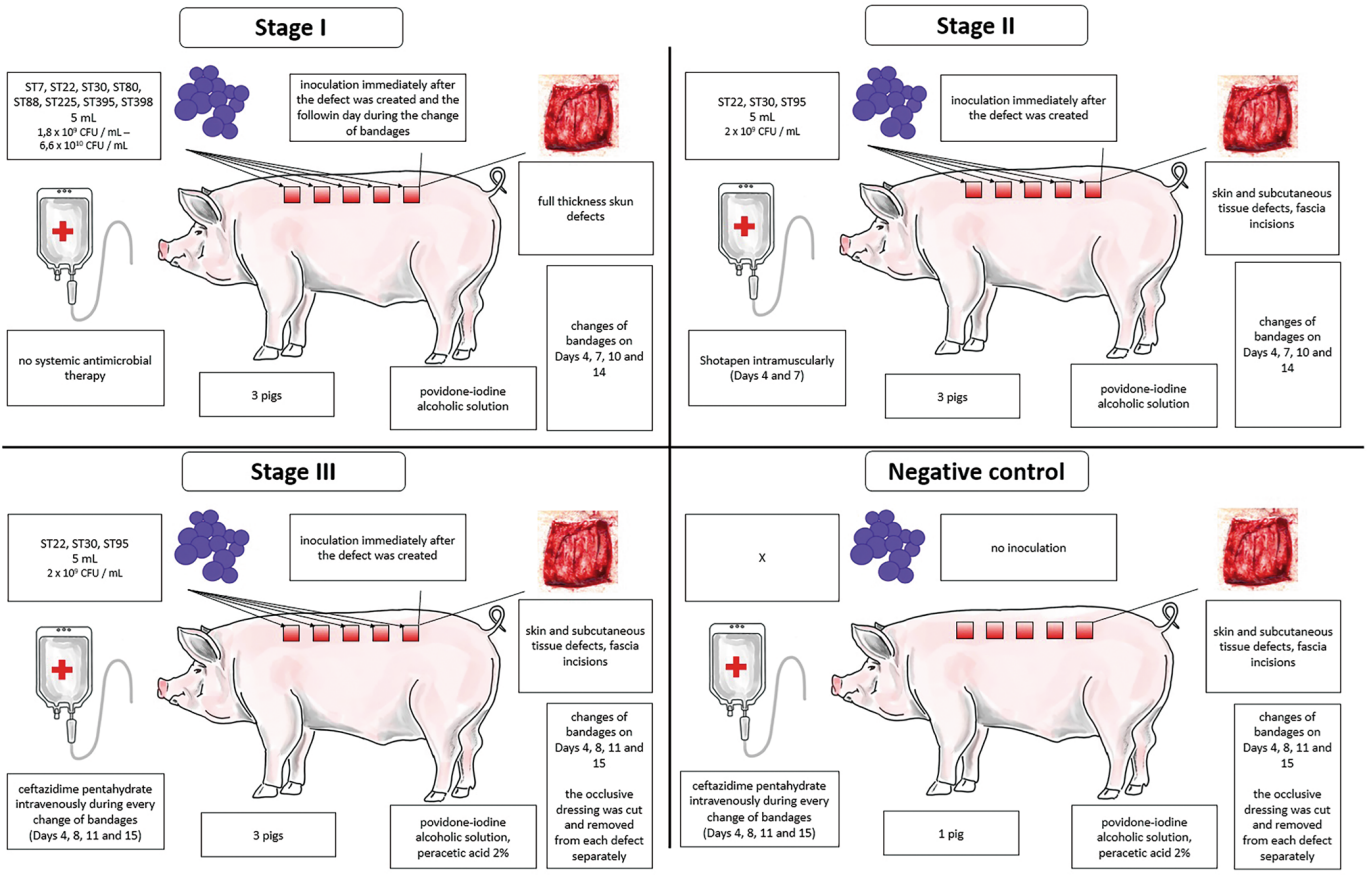
Evolution of protocol stages during the development of skin and subcutaneous tissue, including the negative control.

The experiment was performed in three consecutive stages using three, three, and four pigs, respectively (Figure 1). One pig from stage III was used as a negative control. Female pigs of the hybrid Large White (50%) × Landrace (50%) (Bioprodukt Knapovec a.s., Ústí nad Orlicí, Czech Republic) at the age of 10 weeks were used. The pigs were housed individually in the accredited barrier‐type animal facilities of the Veterinary Research Institute, Brno. The pigs were fed a standard commercial diet (De Heus, Marefy, Czech Republic) twice daily ad libitum and had unlimited access to drinking water.

Premedication and analgesia during the surgical procedure were provided with subcutaneous administration of butorphanol (0.1 mg/kg body weight [BW]). General anesthesia was administered intramuscularly using a combination of tiletamine/zolazepam (2 mg/kg BW), ketamine (2 mg/kg BW), and xylazine (2 mg/kg BW) and was maintained with intravenous propofol (8–15 mg/kg BW).

**FIGURE 2:**
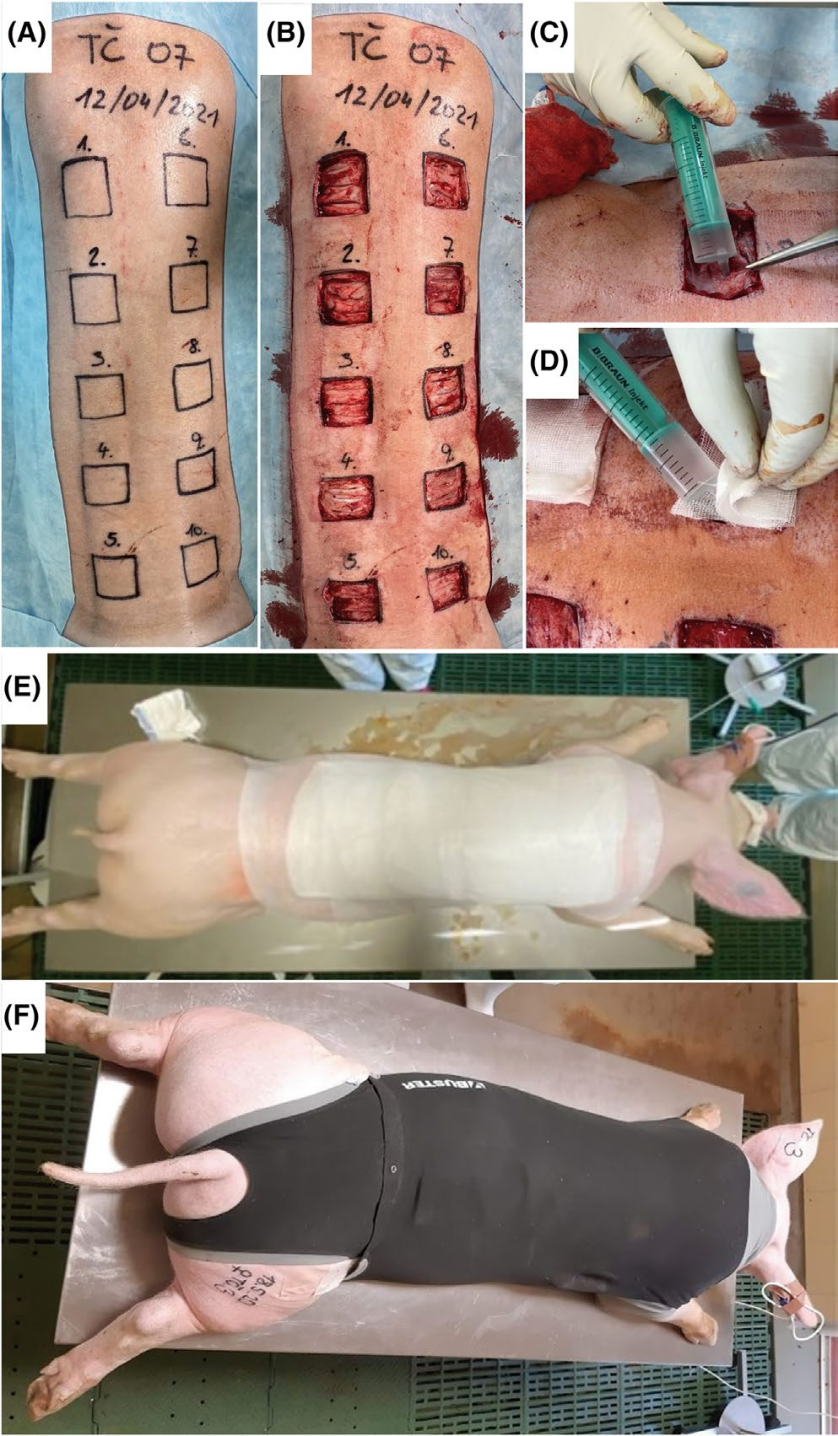
(A) After defects structure design, (B) excisions of the skin and subcutaneous tissue augmented by several fascial incisions were performed. The bacterial inoculum (2 × 10^9^ CFU/mL) was applied immediately after the surgery. (C) One milliliter was inserted deep into the fascial incisions and (D) the remaining 4 mL was applied diffusely into sterile gauzes covering the defect. (E) Occlusive dressing was fixed, and (F) the pigs were dressed in an elastic dress.

In stage I, 5 × 5‐cm full‐thickness skin defects were surgically created on the pigs' backs after antisepsis (povidone–iodine alcoholic solution). The individual skin defects were spaced at least 2 cm apart. In each pig, 10 such skin defects were created. An amount of 5 mL of the respective bacterial inoculum was applied diffusely into the sterile gauzes that individually covered skin defects. Subsequently, all the wounds in each pig were covered together with an occlusive transparent bandage, Hydrofilm (Hartmann‐Rico, Veverská Bítýška, Czech Republic), and sterile gauze. Finally, the pigs were dressed in an elastic dress (Figure 2F). Immediately after surgery, the second analgesic drug, meloxicam (0.1 mg/kg BW), was administered subcutaneously and then continued at a dose of 0.1 mg/kg BW once a day for 3 days.

On days 4, 7, 10, and 14, wound dressings were changed again. After the removal of the dressings and antisepsis (povidone–iodine alcoholic solution), a filter paper imprint technique (FPIT) from each wound was made. Tissue samples from three randomly selected skin defects of each strain (ST22, ST30, and ST395) were taken from each pig using a skin biopsy punch (Medplus, Czech Republic) for microbiological (8 mm diameter) and molecular biological (4 mm diameter) examinations. Tissue samples for histopathological examination were taken using excisional biopsy with a scalpel. At the same time, photographs were taken of each wound. The defects were again covered with sterile gauze and occlusive dressing, and the pigs were dressed in elastic dresses.

Based on the results of stage I, the following changes were made in the protocol during stage II: fascial incisions inside the defects were performed; only three bacterial strains were used; bacterial inoculum was applied only once during the surgery (Figure 2), and Shotapen (combination of antibiotics benzathine benzylpenicillin 10 mg/kg BW, procaine benzylpenicillin 10 mg/kg BW, and dihydrostreptomycin sulfate 20.5 mg/kg BW) was administered intramuscularly during the change of bandages on days 4 and 7.

**FIGURE 3:**
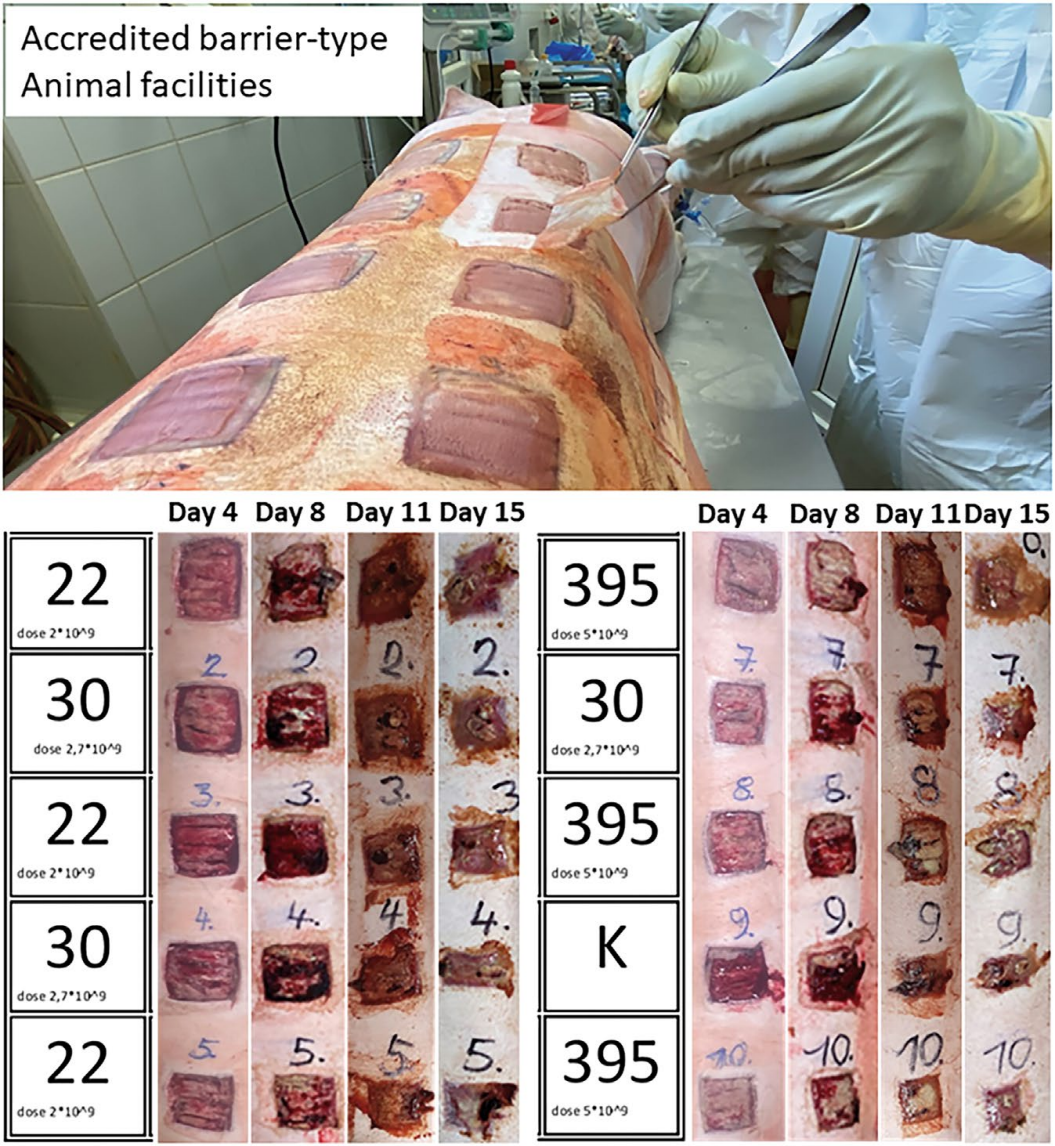
Animal surgery was performed in an accredited barrier‐type animal facility where infectious material could be used. Wound dressings were changed on days 4, 8, 11, and 15. The occlusive dressing was incised and removed from each defect separately to prevent contamination from adjacent wounds. Before each dressing change, photo documentation was carried out as a whole and then in detail for individual wounds. To avoid changing the photo, a map of the wounds was made before each attempt, which was photographed together with the defects. The numbers “22,” “30,” and “395” indicate the sequence types of bacterial strains that were inoculated into the individual wound. The letter “K” indicates a negative control, which means a defect without inoculation of any bacterial strain.

Based on the results of stages I and II, the following changes were made during stage III: to minimize the risk of unwanted contamination, 2% peracetic acid was added as an antiseptic agent, and ceftazidime pentahydrate (2 g) was administered intravenously during every change of bandages (days 4, 8, 11, and 15). Additionally, to minimize the contamination of the air with aerosol released from the dressing during its removal, the occlusive dressing was cut into stripes and removed individually for each wound (Figure 3).

Animal experiments were carried out in accordance with the recommendations of the Czech guidelines for animal experimentation and were approved by the Branch Commission for Animal Welfare of the Ministry of Agriculture of the Czech Republic (approval protocol number: MZe 2223 and file number: 8807/2021‐MZE‐18134).

### 2.4 Identification of bacteria from filter paper imprint technique

The FPIT allows identification and semiquantitative estimation of the amount of microorganisms present on the wound surface. Collected samples were applied to the blood agar (Columbia Agar base, Oxoid, UK, with 5% sheep blood) and blood agar containing 10% NaCl (Oxoid, UK). The culture media were incubated at 37°C for 24 h and then visually evaluated. The results were rated as follows: rating 0 = 0 CFU/25 cm^2^, rating + = 1–10 CFU/25 cm^2^, rating ++ = 11–100 CFU/25 cm^2^, rating +++ = 101–1000 CFU/25 cm^2^, rating ++++ = >1000 CFU/25 cm^2^.

### 2.5 Quantification and identification of bacteria in the tissue sample

Tissue samples taken from the defect base were placed in 2 mL of sterile phosphate‐buffered saline and homogenized (Stomacher 80 Biomaster, Seward, UK). Serial 10‐fold dilutions of the homogenate were plated onto blood agar and blood agar supplemented with 10% NaCl. Plates were incubated at 37°C overnight, the CFU counts enumerated, and CFU per gram of the tissue was calculated. Colonies were subsequently analyzed using mass spectrometry Matrix‐assisted laser desorption/ionization‐time of flight (MALDI‐TOF MS) to verify the expected bacterial species.

### 2.6 Histopathological evaluation

The tissue samples were fixed for 24 h in a 10% formaldehyde solution. The material was standardly processed, and the samples were embedded into paraffin blocks. Afterward, they were cut on a Rotary Microtome (Thermo Fisher Scientific, Austria). Serial slices were placed into standard slides and stained with hematoxylin and eosin (Bamed s.r.o., Czech Republic). The prepared slides were evaluated with an Olympus BX53 microscope (Olympus, Japan). Individual classification scores evaluating the dermis, scabs, edema, inflammation, fibrotization, and necrosis were prepared^17^; (Figures [Link], [Link]). Mann–Whitney test was used for statistical analysis. The histopathological evaluation of the samples is shown in the table (Figure 6).

### 2.7 Gene expression

Stabilized tissues were homogenized in TRI Reagent RT (Molecular Research Center, Cincinnati, Ohio, USA) and stored at −70°C. RNA was extracted by phase separation process, further purified using the NucleoSpin RNA Plus Mini Kit (Macherey‐Nagel, Düren, Germany) and reversely transcribed using the Luna Script RT SuperMix Kit (NEB, Ipswich, MA, USA). Triplicated reactions were performed using LightCycler 480 instrument (Roche, Basel, Switzerland) according to the manufacturer instructions—one reaction (3 μL) contained 10 pmol of each of the two primers (Generi Biotech, Hradec Kralove, Czech Republic; Table 2), 1.5 μL of QuantiTect SYBR Green PCR MasterMix (Qiagen, Darmstadt, Germany), and diluted cDNA (0.5 μL). Cycle values (Cq) of the genes of interest (GOI) normalized to the Cq value of the reference TBP1^18^, ^19^ were applied in formula 2(^−ΔCt^).^20^ In graphs, relative gene expression of GOI in infected and control wounds was used. Statistical Mann–Whitney test was employed. Negative controls were in all parts of the assessment.

**TABLE 2:** Gene‐specific primer pairs used in quantitative polymerase chain reaction (qPCR).

Gene	5′ – Forward primer – 3	Primer reference
	5′ – Reverse primer – 3´	Amplicon lenght^1^/Eff.^2^
IL‐1β	GGGACTTGAAGAGAGAAGTGG	Pavlova et al. (2011)^55^
	CTTTCCCTTGATCCCTAAGGT	95/2.025
IL‐8 (CXCL8)	TGAAGAGAACTGAGAAGCAACAACAACAGCAG	Ondrackova et al. (2013)^56^
	TCTTGGGAGCCACGGAGAATGGGT	74/1.956
MMP‐1	GTCTCACCCTTGACCTTCACCAA	Designed in this study
	CACGATGATCTCCCCTGACAAAG	79/2.077
TIMP‐1	CAGGAGTTTCTCATAGCTGGACAAC	Kyrova et al. (2012)^57^
	GTTCCAGGGAGCCACAAAACT	78/2.019
TGF‐β1	TACGCCAAGGAGGTCACCC	von der Hardt et al. (2004)^58^
	CAGCTCTGCCCGAGAGAGC	156/1.959
*TBP1*	AACAGTTCAGTAGTTATGAGCCAGA	Nygard et al. (2007)^59^
	AGATGTTCTCAAACGCTTCG	153/2.118

*Note*: (1) Size of the amplification product in a number of nucleotides for the adopted primer pair was derived using Primer‐BLAST (https://www.ncbi.nlm.nih.gov/tools/primer‐blast/) based on the current nucleotide sequences available in the NCBI GenBank (National Center for Biotechnology Information, PikeBethesda MD, USA).^54^ The primer pairs with at least one of the primers at the mRNA exon–exon junction were preferred to eliminate gDNA detection. TATA‐box binding protein 1 (*TBP1*) was used as a reference housekeeping gene. (2) Efficiency of primer pair evaluated in the study.

Abbreviations: IL‐8, interleukin 8; MMP‐1, matrix metalloproteinase 1; *TBP1*, TATA‐box binding protein 1; TGF‐β1, transforming growth factor β1; TIMP‐1, tissue inhibitor of metalloproteinase 1.

### 2.8 Clinical evaluation of local signs of wound infection

Photographs of all defects were evaluated by eight physicians with extensive clinical experience. Induration, redness, and purulent discharge were the principal evaluated parameters that determined the probability of occurrence of a local inflammatory reaction. The following scale was used: 0 = no signs, 1 = mild signs, 2 = moderate signs, 3 = severe signs (Figure S7). The mean values of the ratings from all observers were calculated and assessed. Mann–Whitney test was used for statistical analysis.

## 3 RESULTS

### 3.1 Model development

During the first phase of the experiment, we tried to induce infection using different doses of inoculum (1.8 × 10^9^–6.6 × 10^10^ CFU/mL). We used eight different sequence types (ST7, ST22, ST30, ST80, ST88, ST225, ST395, and ST398); three of them were also used in the later stages of the experiment (see Table 1). In all cases, significant wound infections developed within 4 days. The only problem was that the pigs' defects began to heal spontaneously, and the bacterial load in the wounds began to significantly decrease. On day 14, apart from the inoculated *S. aureus* strains, there was a minor presence of common skin commensals (e.g., *Staphylococcus hyicus, Staphylococcus chromogenes, Staphylococcus epidermidis, Corynebacterium amycolatum, Acinetobacter lwoffii, Acinetobacter schindleri, Rothia nasimurium, or Staphylococcus simulans*). This condition was probably caused by the development of only a superficial infection, without vertical penetration of the pathogen into deeper structures.

This necessitated the creation of deeper defects that also involved the subcutaneous tissue. The excisions were supported by several incisions through the fascia. Given the satisfactory development of the infection in the first days of stage I, the bacterial inoculum at concentration of 2.0 × 10^9^ CFU/mL was determined to be sufficient. Shotapen was added to the systemic therapy. After these changes were implemented in stage II of the project, we managed to successfully induce skin and soft‐tissue infection persisting for a sufficiently long time (14 days).

Contamination with gram‐negative aerobic bacteria (particularly *Proteus vulgaris* and also *Acinetobacter baumannii* and *Pseudomonas aeruginosa*), however, remained a challenge. We addressed this by employing additional skin antisepsis using 2% peracetic acid and changing Shotapen for ceftazidime as the systemic antibiotic. Results from the microbial, histopathological, and molecular‐biological analyses from the final stage III will be presented later. There were no significant differences in the results for the individual *S. aureus* strains; therefore, the results will be described as the average values of all three strains.

### 3.2 Qualitative and quantitative microbiological evaluation of bacterial loads

*S. aureus* was detected in all samples (10^2^–10^3^ CFU/25 cm^2^ lesion) using the FPIT and MALDI‐TOF identification, corresponding to a rating of +++.

**FIGURE 4:**
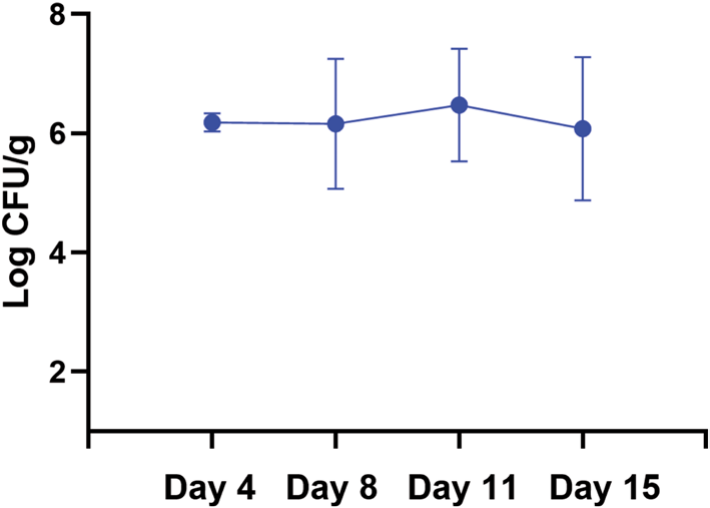
The results of the quantitative evaluation of microorganisms in the tissue samples. The average amount of the methicillin‐resistant *Staphylococcus aureus* (MRSA) strains in inoculated pigs was >10^6^ CFU/g of tissue during the experiment.

The quantification of microorganisms in the tissue samples correlates with the results of the imprint technique. An average quantity of >10^6^ CFU/g (6.2 ± 0.1 log CFU/g) of tissue was found on day 4 after inoculation. An average amount of bacteria (>10^6^ CFU/g) was maintained until day 15 (Figure 4).

These results proved a successful induction of wound infection since day 4 after inoculation. Repeated tissue collection in the following days of the experiment showed that the infection persisted in the tissues till the end of the experiment. At the same time, unwanted contamination by other pathogens was not significant.

### 3.3 Histopathological evaluation

**FIGURE 5:**
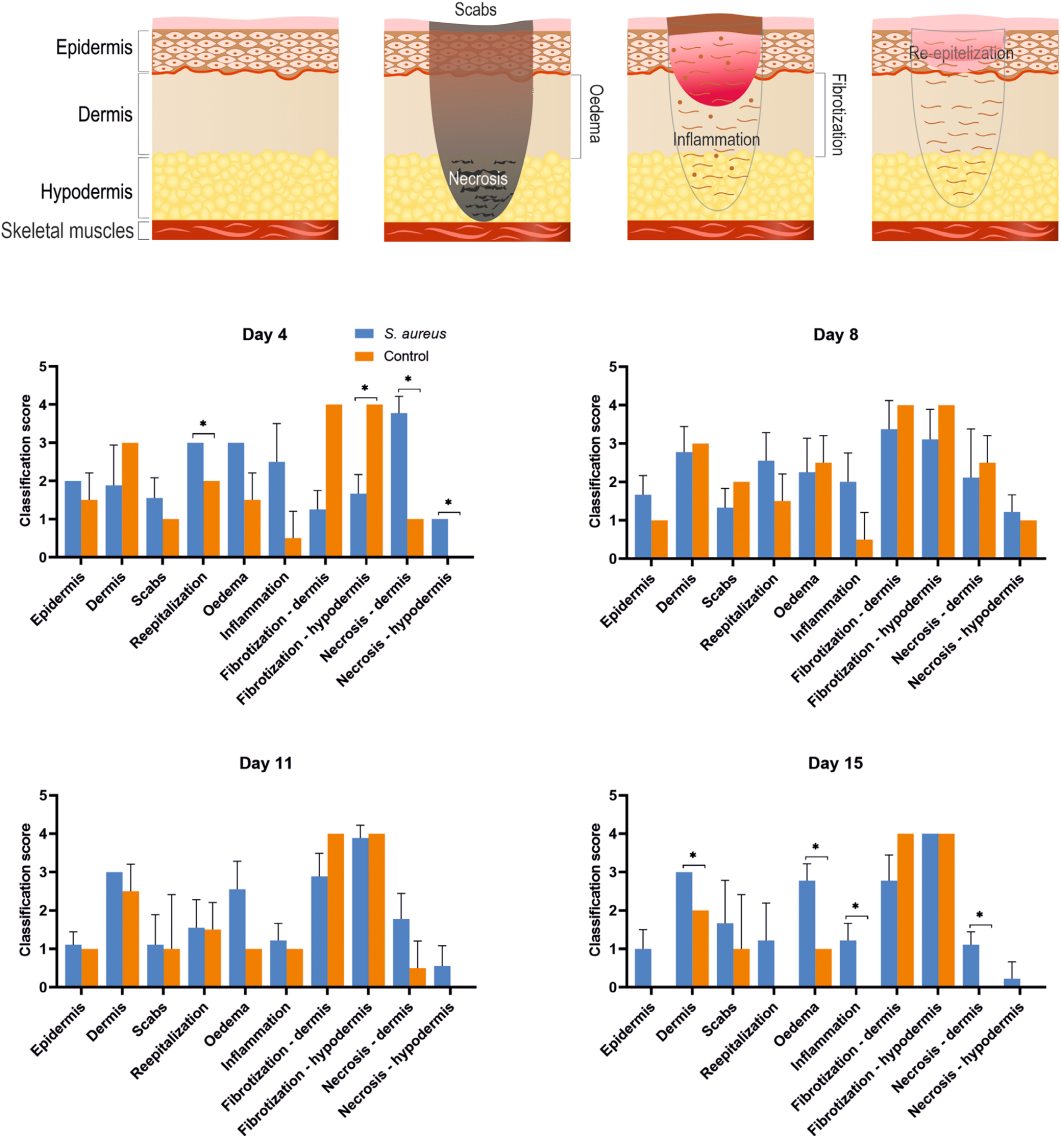
Results of individual classification scores assessing dermis, scab, edema, inflammation, fibrotization, and necrosis. A schematic picture shows all the classified parts of the skin. The individual graphs show the histopathological evaluation of the samples based on the table (Figure S6). Graphs represent the histological analysis found in the wound at 4, 8, 11, and 15 days after induction of infection. Histological analysis data are presented as mean with standard deviation (SD). Significant differences (*p* < 0.05) between infected (blue) and control (orange) wounds are marked with *.

In the initial stages of sampling, there is massive bleeding defects penetrating into the subcutaneous tissue with bacterial load. An intensive soft‐tissue proliferation from the base of the defect could be observed, with the formation of primitive fibrous tissue with scabs and bacterial load. On the periphery, signs of initial reepithelialisation were visible, whereas in the center, edema with necrotic and fibrotic regions, including round cell inflammatory infiltrate, was visible. At the final stage, massive granuloma layered by a continual thin epidermis was visible (Figure 5).

### 3.4 Molecular‐biological evaluation

**FIGURE 6:**
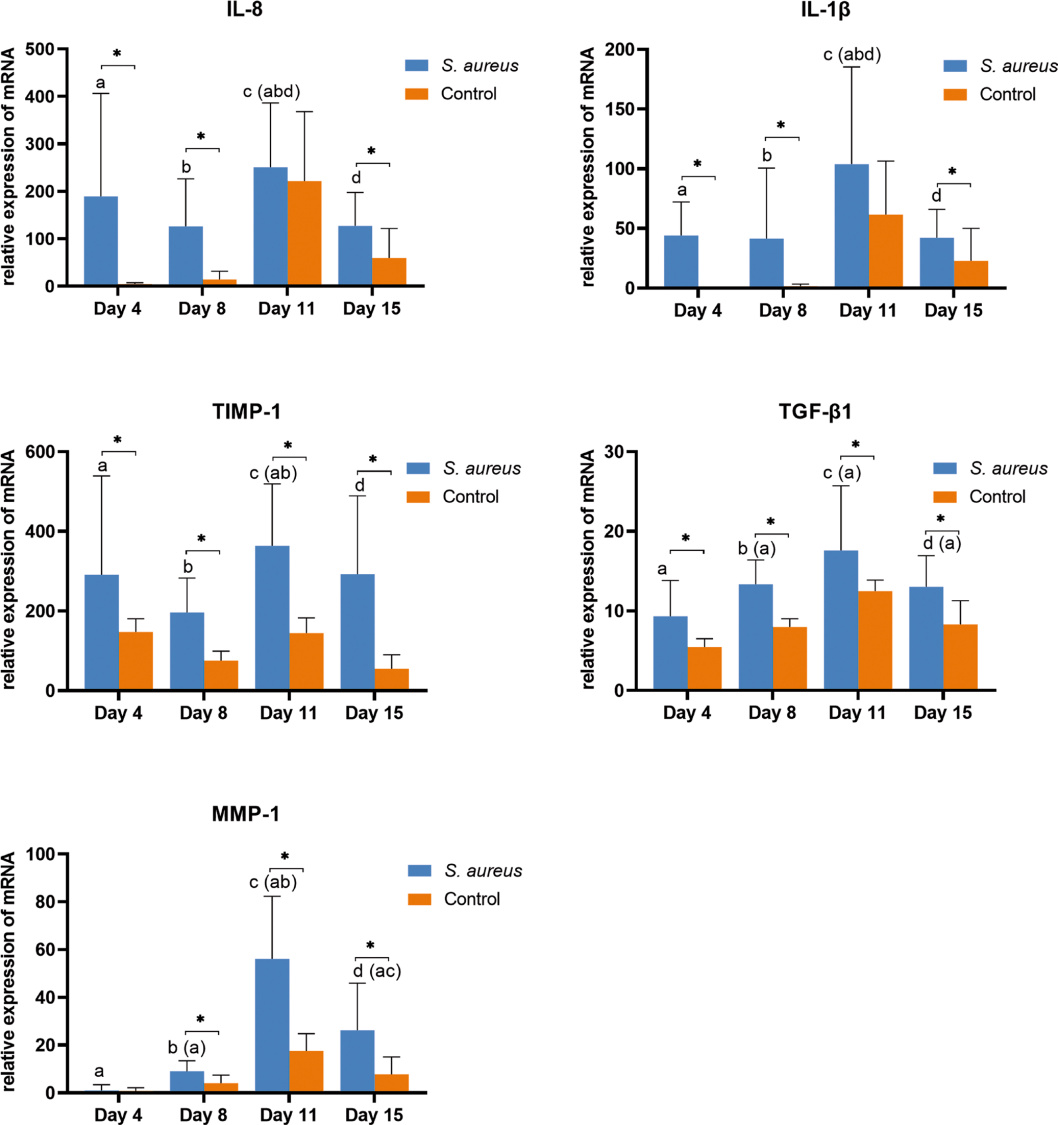
Relative gene expression of selected inflammation and extracellular matrix (ECM)‐related transcripts in infected (blue) and control (orange) wounds. Wound biopsies were measured in real‐time polymerase chain reaction (PCR) using gene‐specific primers on days 4, 8, 11, and 15 after induction of infection (interleukin 8 [IL‐8], IL‐1β, tissue inhibitor of metalloproteinase 1 [TIMP‐1], transforming growth factor β1 [TGF‐β1], matrix metalloproteinase 1 [MMP‐1]). The degree of gene expression is displayed as a fold of housekeeping gene TATA‐box binding protein 1 (*TBP1*) expression. Graphs represent the means of the values with standard deviation (SD). Significant differences (*p* < 0.05) between infected and control wounds are denoted by *. The letters above each column in infected wounds (*Staphylococcus aureus* column) are marked with the same letter (a, b, c, d) in the given collection date. The results of pairwise comparisons between columns that have significant differences (*p* < 0.05) are indicated in parentheses.

Expression of selected genes involved in inflammation and extracellular matrix (ECM) remodeling was observed during wound healing in both infected and uninfected wounds. Gene expression of the inflammatory mediators interleukin 8 (IL‐8) and interleukin 1β (IL‐1β) significantly increased in infected wounds on days 4 and 8 compared to control. No significant difference was observed on day 11. The infection in the infected wounds was more intense, which was also demonstrated by the significantly increased expression of matrix metalloproteinase 1 (MMP‐1), especially on the 11th and 15th days. Transforming growth factor β1 (TGF‐β1) was found to be significantly upregulated compared to control on all days. Tissue inhibitor of metalloproteinase 1 (TIMP‐1) expression changed only slightly over time. TGF‐β1 expression was lower on day 4 compared to other days when gene expression increased presumably in response to tissue fibrosis caused by inflammation (Figure 6).

### 3.5 Clinical evaluation of local signs of wound infection

Purulent discharge was the most pronounced local sign of infection, with maximum on days 8 and 11. The mean value of the rating was 1.82 and 1.97 at these time points. In the following days, the degree of purulent discharge began to gradually decrease.

A very similar situation was observed in the defect induration, with a gradual increase to maxima on days 8 and 11. The average rating value on these days was 0.88 and 0.92. Again, a gradual decrease in the mean value was observed in the following days.

**FIGURE 7:**
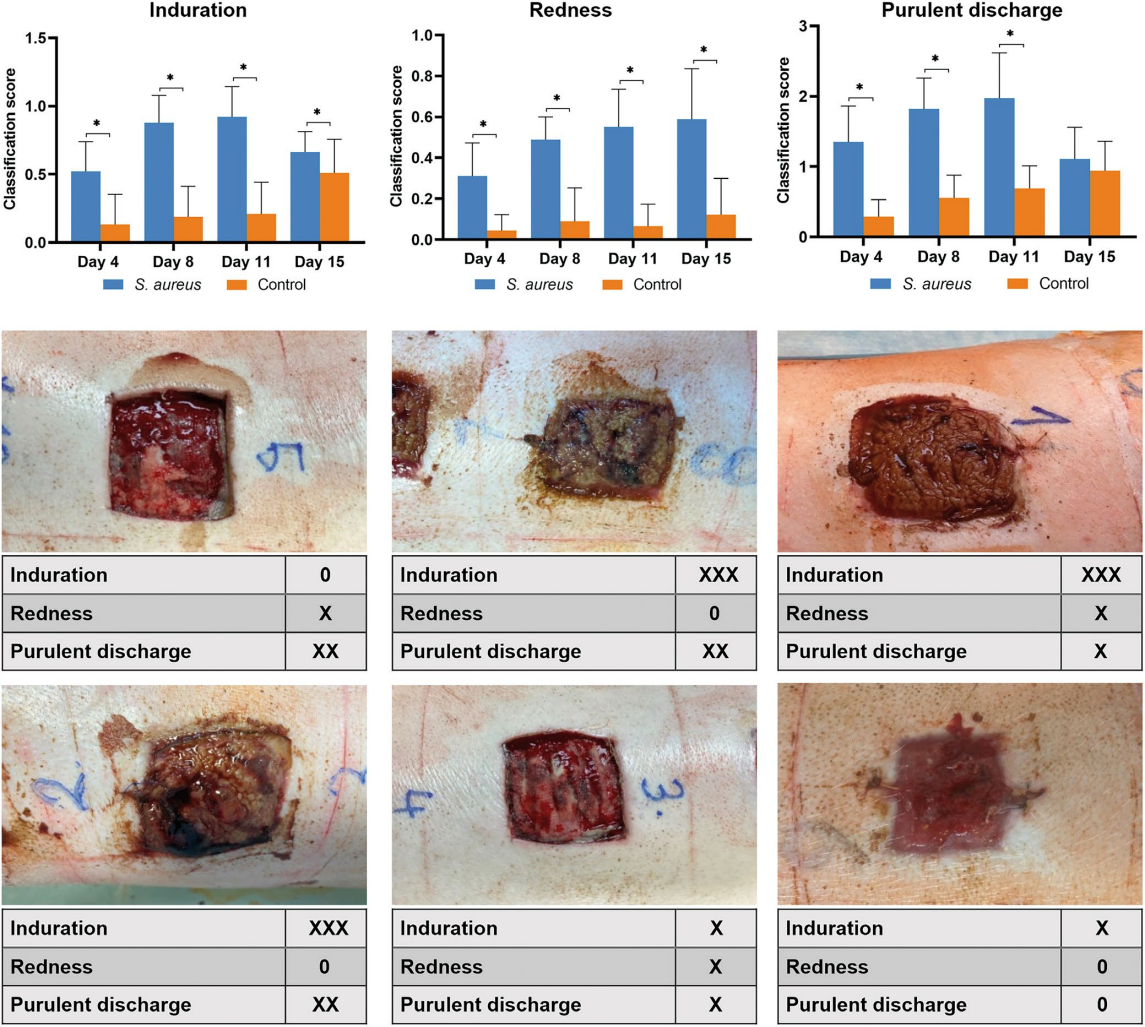
Photographs of all skin and subcutaneous defects were evaluated retrospectively by eight physicians with extensive clinical experience in the field of skin and soft‐tissue wounds of various etiologies. Examples of their evaluation are included in the figure for illustration. The following scale was used: 0 = no signs (0), X = mild signs (1), XX = moderate signs (2), and XXX = severe signs (3). The individual graphs show the clinical evaluation of the samples based on the table (Figure S7). Purulent discharge was the most pronounced local sign of infection, with maxima on days 8 and 11 after the inoculation. A very similar situation was observed in the assessment of defect induration, with a gradual increase to maxima on days 8 and 11 after inoculation. Redness was the least observed local sign of inflammation. Significant differences (*p* < 0.05) between infected (blue) and control (orange) wounds are marked with *.

Redness was the least observable of all evaluated local signs of inflammation. Unlike humans, piglets respond to a microbiologically proven infection with minimal redness around the wound. In our evaluation, the average rating was below 0.6 throughout the experiment, namely 0.31, 0.49, 0.55, and 0.59 on days 4, 8, 11, and 15, respectively (Figure 7).

### 3.6 Negative control evaluation

In the control pig, unwanted contamination was not significant (data not shown). The histopathological changes in the negative control were very similar to those in infected pigs, but they were accelerated. The dynamics of cytokine expression in the negative control were similar to those of the inoculated ones but at significantly lower levels. In the negative control pig, all observed clinical signs of inflammation were significantly less pronounced. Purulent discharge was the most pronounced sign of inflammation. Even on day 15, when the purulent discharge was strongest, it was still graded as 1 (mild discharge).

## 4 DISCUSSION

The multidrug resistance represents a serious medical problem and constitutes a major challenge to the treatment of infectious diseases. There is an urgent need to develop new antimicrobial agents with diverse chemical structures and novel mechanisms of action to overcome the resistance.^14^, ^16^, ^21^ To test novel treatment approaches specifically targeting *S. aureus* infection, it is essential to establish a clinically relevant animal model to validate in vitro results.

Results from stage I revealed that contamination of the wounds with commensal bacteria was the main problem. This precondition was essential for future testing of the efficacy of topical antimicrobials—with microbial contamination, it would be difficult to prove whether the potential eradication of *S. aureus* in the wounds was caused by the therapeutic application of the tested antimicrobial or by competitive inhibition by other microorganisms.

At the beginning of stage III, the concept of antisepsis was modified. Initially, we used povidone–iodine alcoholic solution alone. As its effectiveness was insufficient, we decided to add 2% peracetic acid. This organic peroxide is, owing to its high oxidizing potential, an ideal antimicrobial agent. Similar to hydrogen peroxide, peracetic acid probably denatures proteins and enzymes and increases cell wall permeability by disrupting sulfhydryl and sulfur bonds.^22^ It is broadly effective against microorganisms and is not deactivated by catalases or peroxidases, that is, enzymes that break down hydrogen peroxide.^23^ Several studies demonstrated the high effectiveness of peracetic acid disinfection, especially against gram‐negative bacteria.^24^, ^25^, ^26^

After the antiseptics had evaporated, the occlusive dressing was cut in a way that enabled individual removal from each defect. This procedure has not yet been described in the literature. This measure was taken to minimize the contamination of the air with aerosol released from the dressing during its removal. Airborne microorganisms have repeatedly been proven to have the potential to deposit in surgical wounds and cause infectious complications.^27^, ^28^, ^29^, ^30^, ^31^ The aforementioned antisepsis approach was applied during each change of bandages in stage III of the experiment.

Another necessary alteration of the experimental protocol in stage III concerned an preoperative antibiotic prophylaxis. After successful introduction of the infection, Shotapen was further complemented by ceftazidime pentahydrate. Ceftazidime pentahydrate is a “third‐generation” cephalosporin with activity against gram‐positive and gram‐negative bacteria. It is particularly active against Enterobacterales and is resistant to hydrolysis by most β‐lactamases. On the contrary, its effectiveness against gram‐negative anaerobes is limited, and it is also less effective against some gram‐positive cocci than other newer β‐lactam compounds.^32^ This is, however, not an issue in our case, as no such bacteria were found in the defects.

In infected wounds, there was a complete correlation between all findings: clinical evaluation, study covered bacteriological examination, histopathological assessment of changes associated with process of an inflammation development, and subsequent healing process together with semiquantitative calculation of mRNA expression for genes linked to pro‐ or anti‐inflammatory cytokines. In neither case did the wound heal completely. Despite the stable bacterial load in infections, the surgical wounds are without noticeable infectious complications. The pig model can handle such strong injuries and, unlike humans, can manage the infection. In contrast to sterile inflammation, the inflammatory response induced by infection is associated with the recognition of pathogen‐associated molecular patterns by means of toll‐like receptors in particular.^33^, ^34^ Sterile inflammation activates the immune system using Damage‐associated molecular patterns‐recognizing receptors, like Receptor for Advanced Glycation Endproducts, which is part of homeostatic mechanisms, primarily associated with the removal of destroyed tissue and the subsequent healing of damage.^35^, ^36^ The meaning of this type of inflammation is primarily to eliminate the causing agent, for example, by phagocytosis.

Detailed healing processes, especially in cases of greater tissue damage, involve the replacement of the original tissue with less functional tissue, such as collagen during scar formation. This process is largely controlled by the ratio between the presence of MMPs responsible for the destruction of extracellular proteins^37^ and TIMPs inhibiting action of MMPs.^38^ Unlike MMPs, the expression of TIMPs is more or less constitutive.^39^ This is also evident from our results presented in Figure 5. The activity of MMPs, and especially MMP1 used in our study, increases especially in the phase of angiogenesis and, conversely, decreases again in the phase of remodeling.^38^ This is also consistent with our results, with mRNA expression being higher in infected lesions than in noninfected lesions. Pro‐healing protein TGF‐β1 is responsible for many aspects of reepitelization.^40^ From the result, it can be seen that the infection model has a visible yellow‐white purulent exudate on the 15th day of infection, unlike the control wound, which tends to heal. The difference in wound size at the end of the experiment is also visible. An uninfected wound healed to less than half of its original size, whereas an infected wound healed to a maximum of two‐thirds of its original size (Figure S8).

Several articles describing successful induction of skin infection in an animal model caused by *S. aureus* have been presented in the literature. Most of them were performed on rodents.^41^, ^42^, ^43^, ^44^, ^45^ Due to low cost, easy handling, and the fact that rodents are readily obtainable, it enables researchers to include a relatively large number of animals. This may be the explanation for the sparser literature using pig models, despite greater comparability with human skin.^42^

Jordan et al.^41^ documented guinea pig model of superficial *S. aureus* skin infection. The inoculum was applied onto the intact skin, avoiding any traumatic damage to the skin surface. Mohiti‐Asli et al.^46^ described new porcine model for investigation of treatments of surgical site infection. They performed linear full‐thickness skin incisions down to the fascia of the lumbar muscles, making sure not to perforate or damage the fascia. Nielsen et al.^47^ described a porcine model of subcutaneous *S. aureus* infection. The bacterial inoculum was injected there using a hypodermic needle. All these authors used very similar amounts of pathogen to induce skin and soft‐tissue infection as in our study.

As shown by the stage I results in our porcine model development, if only superficial defects were created in an immunocompetent pig, the animals were able to eradicate the local infection and spontaneously heal the wounds. The infection did not persist in the wound for a sufficient period of time. This was one of the first reasons that led us to the need to create a completely new animal model on which the efficiency of new antimicrobial agents could be adequately assessed.

Zurawski et al.^48^ described a porcine wound model of *A. baumannii* infection using a small dose of cyclophosphamide. No immunosuppressant agents were needed in our approach for creating an animal wound model. Although varying degrees of immunoparalysis can be observed in patients with extensive wounds and a reduction in neutrophil infiltration in aging nonhealing wounds (especially diabetic defects), *S. aureus* infections are not typical in neutropenic patients.^49^

Several porcine models with polymicrobial skin and soft‐tissue infections are described in the literature. Klein et al.^50^ documented a clinically relevant porcine model of a biofilm‐infected wound. They used modified polymicrobial Lubbock chronic wound biofilm consisting of *S. aureus*, *Enterococcus faecalis*, *P. aeruginosa*, and *Bacillus subtilis*. Li et al.^51^ presented swine model of infected soft‐tissue blast injury. In this case, there was a spontaneous development of soft‐tissue infection with a mixture of pathogens, not the development of an infection based on inoculation with a single pathogen.

Contamination of the defects by other bacterial strains has not been evaluated or described in almost any of the previously published porcine infection models. Hirsch et al.^52^ presented a porcine excisional diabetic wound healing model after inoculation with *S. aureus*. Colonization by *Escherichia coli*, *Streptococcus dysgalactiae*, and *Enterococcus faecium* was observed there; however, this coinfection was not treated in any way.^52^ Based on our experience from the first two stages of our model development, the use of such models for testing the effectiveness of novel antimicrobial agents is limited.

We are currently preparing a randomized controlled trial using the biobetters‐containing material for wound MRSA infections and intend to offer it for future commercial/clinical use.^53^

### 4.1 LIMITATIONS OF THE STUDY

During the development of our animal model of skin and soft‐tissue infection caused only by *S. aureus*, several limiting factors appeared. Due to the natural way of life of the pigs (digging, scratching, or rubbing against the walls, etc.), partial contamination of the bandages occurred. The preoperative antibiotic prophylaxis administration can also be considered a limitation and possible interfering factor with the tested antimicrobial substances.

## 5 AUTHOR CONTRIBUTIONS

**Filip Raška:** Data curation; writing – original draft. **Břetislav Lipový:** Conceptualization; data curation; supervision; writing – review and editing. **Šárka Kobzová:** Data curation; formal analysis; investigation; writing – review and editing. **Lukáš Vacek:** Conceptualization; formal analysis; investigation; writing – review and editing. **Rea Jarošová:** Methodology. **Dominika Kleknerová:** Methodology. **Katarína Matiašková:** Formal analysis. **Peter Makovický:** Data curation; formal analysis; methodology; writing – review and editing. **Monika Vícenová:** Methodology. **Edita Jeklová:** Methodology. **Roman Pantůček:** Resources. **Martin Faldyna:** Conceptualization; validation; writing – review and editing. **Lubomir Janda:** Conceptualization; data curation; formal analysis; investigation; supervision; writing – review and editing.

## FUNDING INFORMATION

This study was Supported by the Ministry of Health of the Czech Republic (grants NV19‐05‐00214 and NU22‐05‐00475).

## CONFLICT OF INTEREST STATEMENT

The authors declare no conflict of interest.

## 7 ETHICS STATEMENT

Animal experiments were carried out in accordance with the recommendations of the Czech guidelines for animal experimentation and were approved by the Branch Commission for Animal Welfare of the Ministry of Agriculture of the Czech Republic (approval protocol No. MZe 2223, File number: 8807/2021‐MZE‐18134)

## Supporting information


Figure S1.

Figure S2.

Figure S3.

Figure S4.

Figure S5.

Figure S6.

Figure S7.

Figure S8.

**Figure Captions**.

## References

[1] Ding X , Tang Q , Xu Z , et al. Challenges and innovations in treating chronic and acute wound infections: from basic science to clinical practice. Burns & Trauma. 2022;10:tkac014. doi:10.1093/burnst/tkac014 35611318 PMC9123597

[2] Lipový B , Brychta P , Řihová H , et al. Prevalence of infectious complications in burn patients requiring intensive care: data from a pan‐European study. Epidemiol Mikrobiol Imunol. 2016;65(1):25‐32. English. 27246641

[3] Warner PM , Coffee TL , Yowler CJ . Outpatient burn management. Surg Clin North Am. 2014;94(4):879‐892. doi:10.1016/j.suc.2014.05.009 25085094

[4] Eckmann C , Lawson W , Nathwani D , et al. Antibiotic treatment patterns across Europe in patients with complicated skin and soft‐tissue infections due to meticillin‐resistant *Staphylococcus aureus*: a plea for implementation of early switch and early discharge criteria. Int J Antimicrob Agents. 2014;44(1):56‐64. doi:10.1016/j.ijantimicag.2014.04.007 24928311

[5] Ceballos S , Aspiroz C , Ruiz‐Ripa L , Azcona‐Gutierrez JM , López‐Cerero L , López‐Calleja AI , Álvarez L , Gomáriz M , Fernández M , Torres C , Ezpeleta C , Martín C , Arribas J , Navarro C , Arias A , Fortuño B , Pereira J , Milagro A , Torres L , Soria‐Blanco LM , Canut A , Cordón ML , Megías G , Calvo J , Rezusta A (2019) Study Group of Clinical LA‐MRSA; Study Group of Clinical LA‐MRSA . Multicenter study of clinical non‐β‐lactam‐antibiotic susceptible MRSA strains: genetic lineages and Panton‐valentine leukocidin (PVL) production. Enferm Infecc Microbiol Clin (Engl ed). 37(8), 509–513. English, Spanish. doi: 10.1016/j.eimc.2019.01.015 30879606

[6] Mohamed MA , Nasr M , Elkhatib WF , Eltayeb WN , Elshamy AA , El‐Sayyad GS . Nanobiotic formulations as promising advances for combating MRSA resistance: susceptibilities and post‐antibiotic effects of clindamycin, doxycycline, and linezolid. RSC Adv. 2021;11(63):39696‐39706. doi:10.1039/d1ra08639a 35494109 PMC9044563

[7] Chang S , Sievert DM , Hageman JC , et al. Vancomycin‐resistant Staphylococcus aureus investigative team. Infection with vancomycin‐resistant Staphylococcus aureus containing the vanA resistance gene. N Engl J Med. 2003;348(14):1342‐1347. doi:10.1056/NEJMoa025025 12672861

[8] Marra A . Animal Models for Drug Development for MRSA. Methods Mol Biol. 2020;2069:253‐266. doi:10.1007/978-1-4939-9849-4_17 31523778

[9] Sullivan TP , Eaglstein WH , Davis SC , Mertz P . The pig as a model for human wound healing. Wound Repair Regen. 2001;9(2):66‐76. doi:10.1046/j.1524-475x.2001.00066.x 11350644

[10] Dahiya P . Burns as a model of SIRS. Front Biosci (Landmark ed). 2009;14(13):4962‐4967. doi:10.2741/3580 19482598

[11] Davidson JM . Animal models for wound repair. Arch Dermatol Res. 1998;290(Suppl):S1‐S11. doi:10.1007/pl00007448 9710378

[12] Wong VW , Sorkin M , Glotzbach JP , Longaker MT , Gurtner GC . Surgical approaches to create murine models of human wound healing. J Biomed Biotechnol. 2010;2011:969618. doi:10.1155/2011/969618 21151647 PMC2995912

[13] Meyer W , Schwarz R , Neurand K . The skin of domestic mammals as a model for the human skin, with special reference to the domestic pig. Curr Probl Dermatol. 1978;7:39‐52. doi:10.1159/000401274 752456

[14] Vacek L , Kouřilová M , Kobzová Š , Janda L . Antimicrobial effect of endolysins LYSDERM‐S and LYSDERM‐T1 and endolysin‐ubiquitin combination on methicillin‐resistant *Staphylococcus aureus* . Biologia. 2022;78:601‐608. doi:10.1007/s11756-022-01282-6

[15] Winstel V , Sanchez‐Carballo P , Holst O , Xia G , Peschel A . Biosynthesis of the unique wall teichoic acid of *Staphylococcus aureus* lineage ST395. MBio. 2014;5(2):e00869. doi:10.1128/mBio.00869-14 24713320 PMC3993852

[16] Vacek L , Kobzová Š , Čmelík R , Pantůček R , Janda L . Enzybiotics LYSSTAPH‐S and LYSDERM‐S as potential therapeutic agents for chronic MRSA wound infections. Antibiotics (Basel). 2020;9(8):519. doi:10.3390/antibiotics9080519 32824115 PMC7459665

[17] Makovický P , Jeklová E , Lipový B , et al. A scoring system for histological evaluation of skin during *Staphylococcus aureus* infections. Biologia. 2024;79:917‐925. doi:10.1007/s11756-023-01567-4

[18] Andersen CL , Jensen JL , Ørntoft TF . Normalization of real‐time quantitative reverse transcription‐PCR data: a model‐based variance estimation approach to identify genes suited for normalization, applied to bladder and colon cancer data sets. Cancer Res. 2004;64(15):5245‐5250. doi:10.1158/0008-5472.CAN-04-0496 15289330

[19] Bustin SA , Benes V , Garson JA , et al. The MIQE guidelines: minimum information for publication of quantitative real‐time PCR experiments. Clin Chem. 2009;55(4):611‐622. doi:10.1373/clinchem.2008.112797 19246619

[20] Livak KJ , Schmittgen TD . Analysis of relative gene expression data using real‐time quantitative PCR and the 2(‐Delta C(T)) method. Methods. 2001;25(4):402‐408. doi:10.1006/meth.2001.1262 11846609

[21] Malasala S , Ahmad MN , Akunuri R , et al. Synthesis and evaluation of new quinazoline‐benzimidazole hybrids as potent anti‐microbial agents against multidrug resistant *Staphylococcus aureus* and *mycobacterium tuberculosis* . Eur J Med Chem. 2021;212:112996. doi:10.1016/j.ejmech.2020.112996 33190958

[22] Gerald MDG . Block's Disinfection, Sterilization, and Preservation. Sixth ed. Wolters Kluwer Health; 2021. https://search‐ebscohost‐com.ezproxy.muni.cz/login.aspx?direct=true&AuthType=ip,cookie,uid&db=nlebk&AN=3051937&lang=cs&site=eds‐live&scope=site

[23] Gad SC . Peracetic acid. Encyclopedia of Toxicology. Academic Press; 2014:788‐790. doi:10.1016/B978-0-12-386454-3.01197-0

[24] Bridier A , Briandet R , Thomas V , Dubois‐Brissonnet F . Comparative biocidal activity of peracetic acid, benzalkonium chloride and ortho‐phthalaldehyde on 77 bacterial strains. J Hosp Infect. 2011;78(3):208‐213. doi:10.1016/j.jhin.2011.03.014 21664534

[25] Subha N , Prabhakar V , Koshy M , Abinaya K , Prabu M , Thangavelu L . Efficacy of peracetic acid in rapid disinfection of Resilon and gutta‐percha cones compared with sodium hypochlorite, chlorhexidine, and povidone‐iodine. J Endod. 2013;39(10):1261‐1264. doi:10.1016/j.joen.2013.06.022 24041388

[26] Chhetri RK , Sanchez DF , Lindholst S , et al. Disinfection of hospital‐derived antibiotic‐resistant bacteria at source using peracetic acid. Journal of Water Processing Engineering. 2022;45:102507. doi:10.1016/j.jwpe.2021.102507

[27] Whyte W , Hodgson R , Tinkler J . The importance of airborne bacterial contamination of wounds. J Hosp Infect. 1982;3(2):123‐135. doi:10.1016/0195-6701(82)90004-4 6181129

[28] Friberg B , Friberg S , Burman LG . Correlation between surface and air counts of particles carrying aerobic bacteria in operating rooms with turbulent ventilation: an experimental study. J Hosp Infect. 1999a;42(1):61‐68. doi:10.1053/jhin.1998.0542 10363212

[29] Friberg B , Friberg S , Burman LG . Inconsistent correlation between aerobic bacterial surface and air counts in operating rooms with ultra clean laminar air flows: proposal of a new bacteriological standard for surface contamination. J Hosp Infect. 1999b;42(4):287‐293. doi:10.1053/jhin.1998.0598 10467542

[30] Friberg B , Friberg S , Ostensson R , Burman LG . Surgical area contamination–comparable bacterial counts using disposable head and mask and helmet aspirator system, but dramatic increase upon omission of head‐gear: an experimental study in horizontal laminar air‐flow. J Hosp Infect. 2001;47(2):110‐115. doi:10.1053/jhin.2000.0909 11170774

[31] Knobben BA , van Horn JR , van der Mei HC , Busscher HJ . Evaluation of measures to decrease intra‐operative bacterial contamination in orthopaedic implant surgery. J Hosp Infect. 2006;62(2):174‐180. doi:10.1016/j.jhin.2005.08.007 16343691

[32] Gentry LO . Antimicrobial activity, pharmacokinetics, therapeutic indications and adverse reactions of ceftazidime. Pharmacotherapy. 1985;5(5):254‐267. doi:10.1002/j.1875-9114.1985.tb03424.x 3906585

[33] Chen L , DiPietro LA . Toll‐like receptor function in acute wounds. Adv Wound Care. 2017;6(10):344‐355. doi:10.1089/wound.2017.0734 PMC5649397 29062591

[34] Sun L , Liu W , Zhang LJ . The role of toll‐like receptors in skin host defense, psoriasis, and atopic dermatitis. J Immunol Res. 2019;2019:1824624. doi:10.1155/2019/1824624 31815151 PMC6877906

[35] Chen GY , Nuñez G . Sterile inflammation: sensing and reacting to damage. Nat Rev Immunol. 2010;10(12):826‐837. doi:10.1038/nri2873 21088683 PMC3114424

[36] Gong T , Liu L , Jiang W , Zhou R . DAMP‐sensing receptors in sterile inflammation and inflammatory diseases. Nat Rev Immunol. 2020;20(2):95‐112. doi:10.1038/s41577-019-0215-7 31558839

[37] Caley MP , Martins VL , O'Toole EA . Metalloproteinases and wound healing. Adv Wound Care. 2015;4(4):225‐234. doi:10.1089/wound.2014.0581 PMC4397992 25945285

[38] Kandhwal M , Behl T , Singh S , et al. Role of matrix metalloproteinase in wound healing. Am J Transl Res. 2022;14(7):4391‐4405. 35958464 PMC9360851

[39] Stetler‐Stevenson WG . Tissue inhibitors of metalloproteinases in cell signaling: metalloproteinase‐independent biological activities. Sci Signal. 2008;1(27):re6. doi:10.1126/scisignal.127re6 18612141 PMC2493614

[40] Ramirez H , Patel SB , Pastar I . The role of TGFβ signaling in wound epithelialization. Adv Wound Care. 2014;3(7):482‐491. doi:10.1089/wound.2013.0466 PMC4086377 25032068

[41] Jordan WE . The experimental induction of superficial cutaneous infection in Guinea pigs. J Invest Dermatol. 1970;55(2):149‐152. doi:10.1111/1523-1747.ep12291783 5489495

[42] Ghaemi EO , Khorshidi D , Moradi A , et al. The efficacy of ethanolic extract of lemon verbena on the skin infection due to *Staphylococcus aureus* in an animal model. Pak J Biol Sci. 2007;10(22):4132‐4135. doi:10.3923/pjbs.2007.4132.4135 19090293

[43] Prabhakara R , Foreman O , De Pascalis R , et al. Epicutaneous model of community‐acquired *Staphylococcus aureus* skin infections. Infect Immun. 2013;81(4):1306‐1315. doi:10.1128/IAI.01304-12 23381997 PMC3639601

[44] Mohamed MF , Seleem MN . Efficacy of short novel antimicrobial and anti‐inflammatory peptides in a mouse model of methicillin‐resistant *Staphylococcus aureus* (MRSA) skin infection. Drug Des Devel Ther. 2014;8:1979‐1983. doi:10.2147/DDDT.S72129 PMC4207544 25378910

[45] Malachowa N , Kobayashi SD , Lovaglio J , DeLeo FR . Mouse model of *Staphylococcus aureus* skin infection. Methods Mol Biol. 2019;1960:139‐147. doi:10.1007/978-1-4939-9167-9_12 30798528

[46] Mohiti‐Asli M , Risselada M , Jacob M , Pourdeyhimi B , Loboa EG . Creation and evaluation of new porcine model for investigation of treatments of surgical site infection. Tissue Eng Part C Methods. 2017;23(11):795‐803. doi:10.1089/ten.TEC.2017.0024 28750575 PMC5689126

[47] Nielsen OL , Mellergaard M , Frees D , et al. A porcine model of subcutaneous *Staphylococcus aureus* infection: a pilot study. APMIS. 2022;130(7):359‐370. doi:10.1111/apm.13101 33644910

[48] Zurawski DV , Black CC , Alamneh YA , et al. A porcine wound model of *Acinetobacter baumannii* infection. Adv Wound Care (New Rochelle). 2019;8(1):14‐27. doi:10.1089/wound.2018.0786 30705786 PMC6350066

[49] Su Y , Richmond A . Chemokine regulation of neutrophil infiltration of skin wounds. Adv Wound Care (New Rochelle). 2015;4(11):631‐640. doi:10.1089/wound.2014.0559 26543677 PMC4620531

[50] Klein P , Sojka M , Kucera J , et al. A porcine model of skin wound infected with a polybacterial biofilm. Biofouling. 2018;34(2):226‐236. doi:10.1080/08927014.2018.1425684 29405092

[51] Li J , Topaz M , Xun W , et al. New swine model of infected soft tissue blast injury. The Journal of Trauma and Acute Care Surgery. 2012;73(4):908‐913. doi:10.1097/TA.0b013e318253b592 22710779

[52] Hirsch T , Spielmann M , Zuhaili B , et al. Enhanced susceptibility to infections in a diabetic wound healing model. BMC Surg. 2008;8:5. doi:10.1186/1471-2482-8-5 18312623 PMC2276479

[53] Raška F , Lipový B , Holoubek J , et al. Promising effect of topical antimicrobial 'biobetters' against methicillin‐resistant *Staphylococcus aureus* strains. Burns. 2022;48(7):1770‐1772. doi:10.1016/j.burns.2022.08.011 36089543

[54] Ye J , Coulouris G , Zaretskaya I , Cutcutache I , Rozen S , Madden T . Primer‐BLAST: a tool to design target‐specific primers for polymerase chain reaction. BMC Bioinformatics. 2012;13:134. doi:10.1186/1471-2105-13-134 22708584 PMC3412702

[55] Pavlova B , Volf J , Ondrackova P , et al. SPI‐1‐encoded type III secretion system of *salmonella enterica* is required for the suppression of porcine alveolar macrophage cytokine expression. Vet Res. 2011;42(1):16. doi:10.1186/1297-9716-42-16 21314975 PMC3037896

[56] Ondrackova P , Kovaru H , Kovaru F , Leva L , Faldyna M . Adenosine modulates LPS‐induced cytokine production in porcine monocytes. Cytokine. 2013;61(3):953‐961. doi:10.1016/j.cyto.2012.12.026 23388525

[57] Kyrova K , Stepanova H , Rychlik I , Faldyna M , Volf J . SPI‐1 encoded genes of *Salmonella Typhimurium* influence differential polarization of porcine alveolar macrophages *in vitro* . BMC Vet Res. 2012;8:115. doi:10.1186/1746-6148-8-115 22817641 PMC3441223

[58] von der Hardt K , Kandler MA , Fink L , et al. High frequency oscillatory ventilation suppresses inflammatory response in lung tissue and microdissected alveolar macrophages in surfactant depleted piglets. Pediatr Res. 2004;55(2):339‐346. doi:10.1203/01.PDR.0000106802.55721.8A 14663153

[59] Nygard AB , Jørgensen CB , Cirera S , Fredholm M . Selection of reference genes for gene expression studies in pig tissues using SYBR green qPCR. BMC Mol Biol. 2007;8:67. doi:10.1186/1471-2199-8-67 17697375 PMC2000887

