# Supplementary figures and images for: Development of a porcine model of skin and soft‐tissue infection caused by Staphylococcus aureus, including methicillin‐resistant strains suitable for testing topical antimicrobial agents
Source: Animal Model Exp Med. 2024 Oct 31;8(3):544–57. doi: 10.1002/ame2.12495 (PMC11904108; doi:10.1002/ame2.12495)

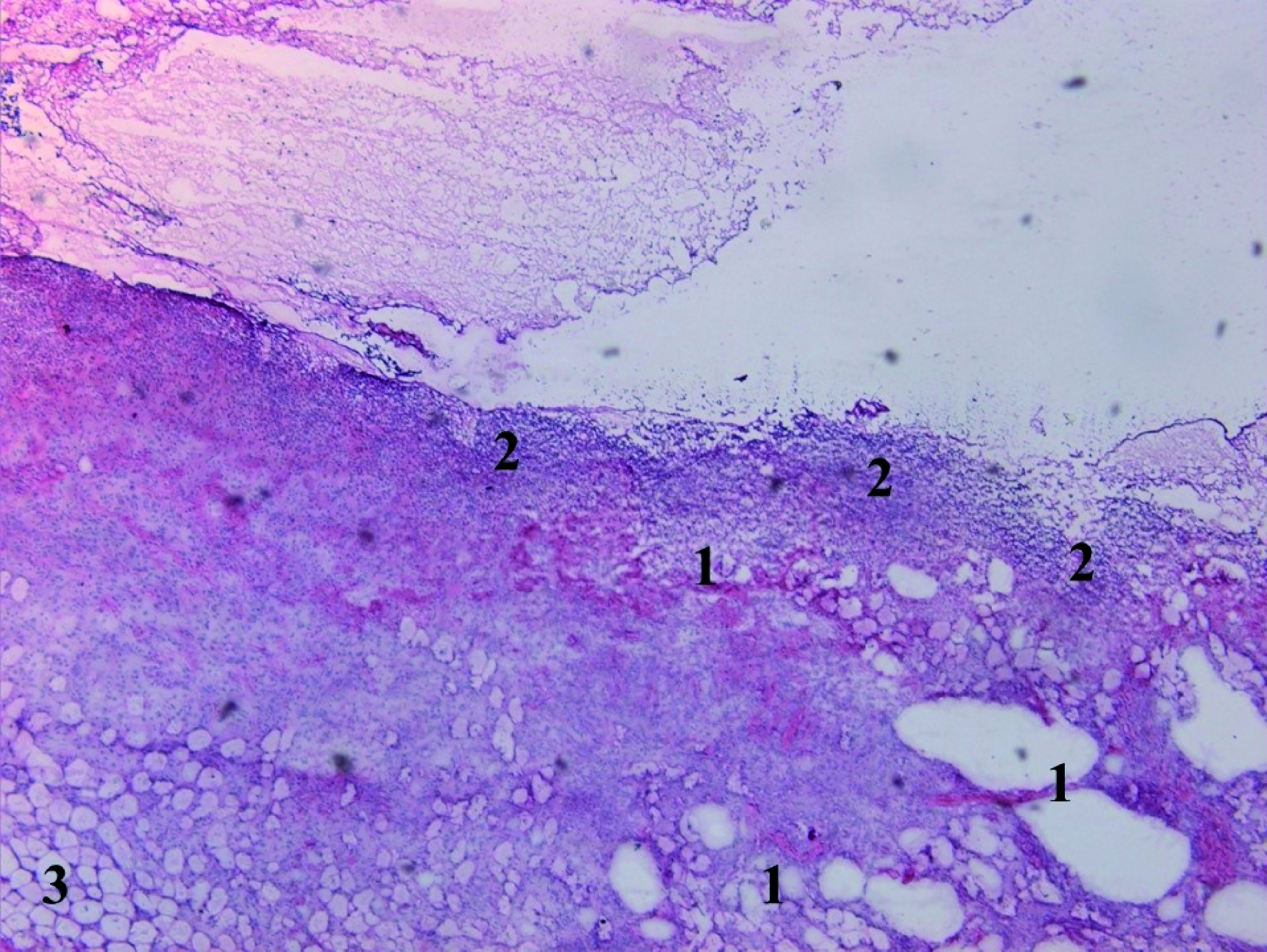

Supplement: Supplementary file 2 — Figure S2. [file AME2-8-544-s006.tif]

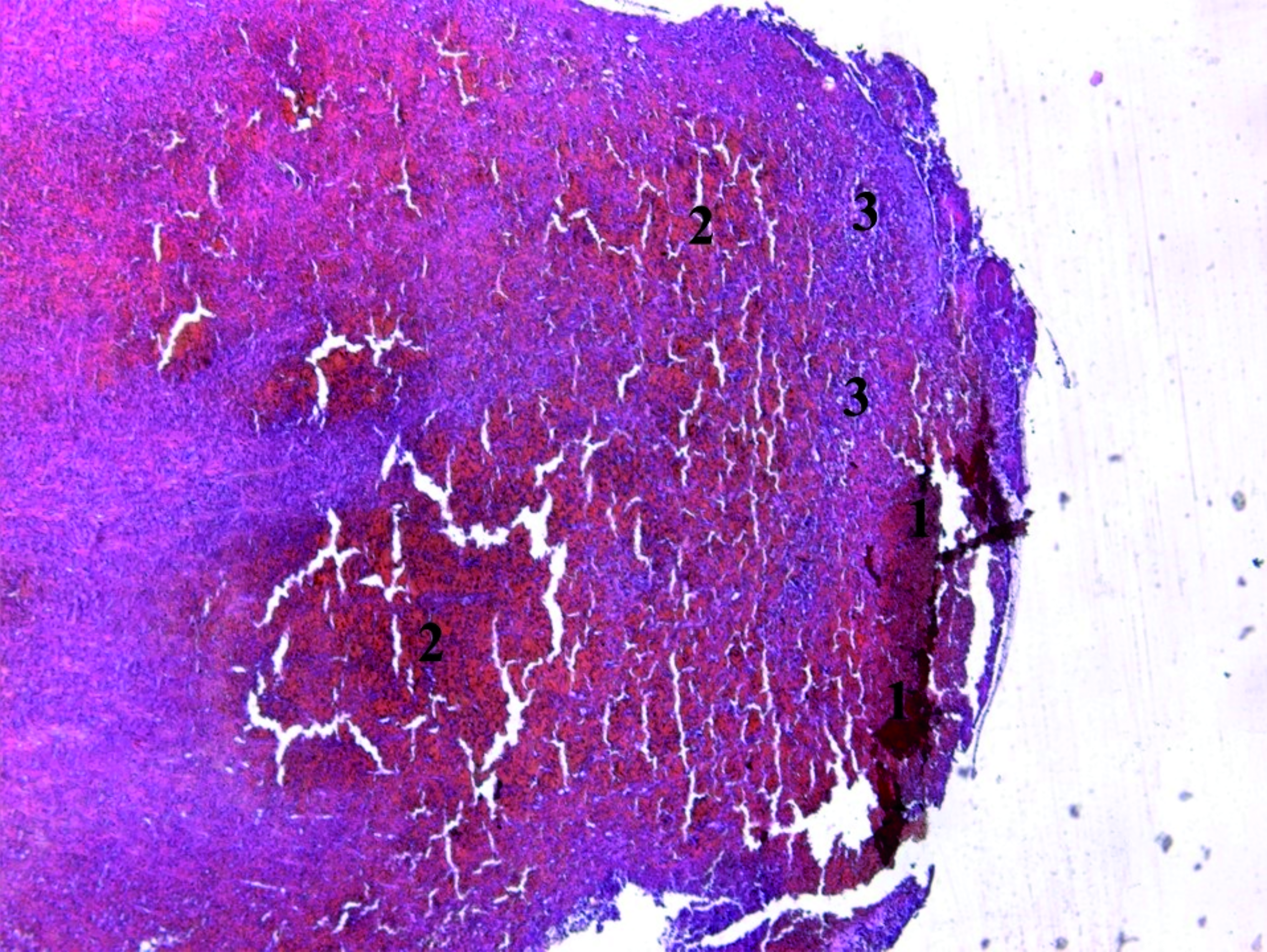

Supplement: Supplementary file 5 — Figure S5. [file AME2-8-544-s007.tif]

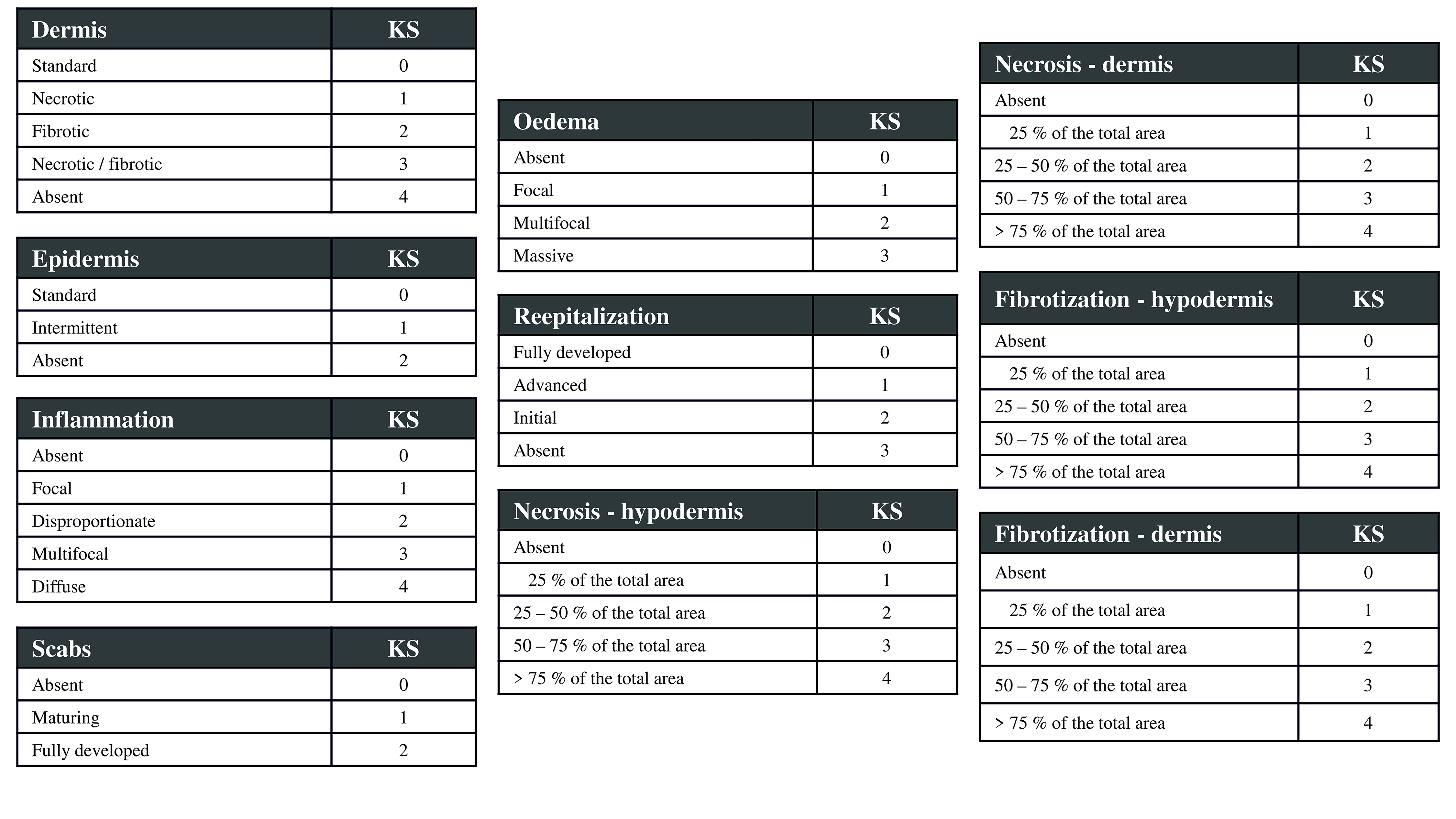

Supplement: Supplementary file 6 — Figure S6. [file AME2-8-544-s004.tif]

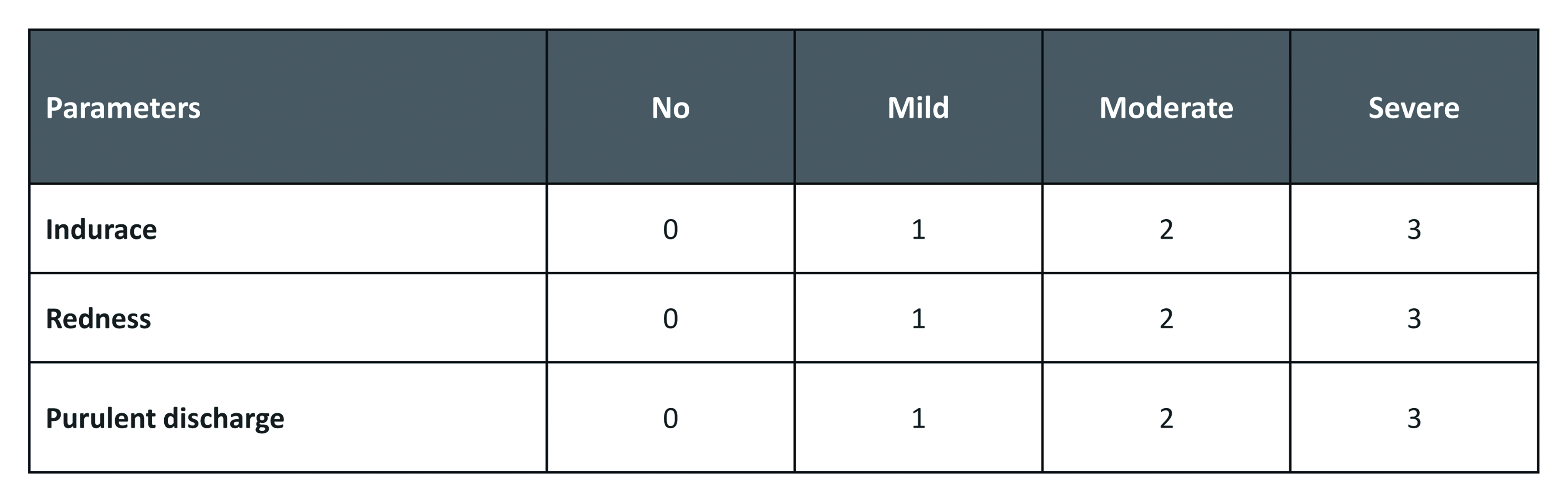

Supplement: Supplementary file 7 — Figure S7. [file AME2-8-544-s002.tif]

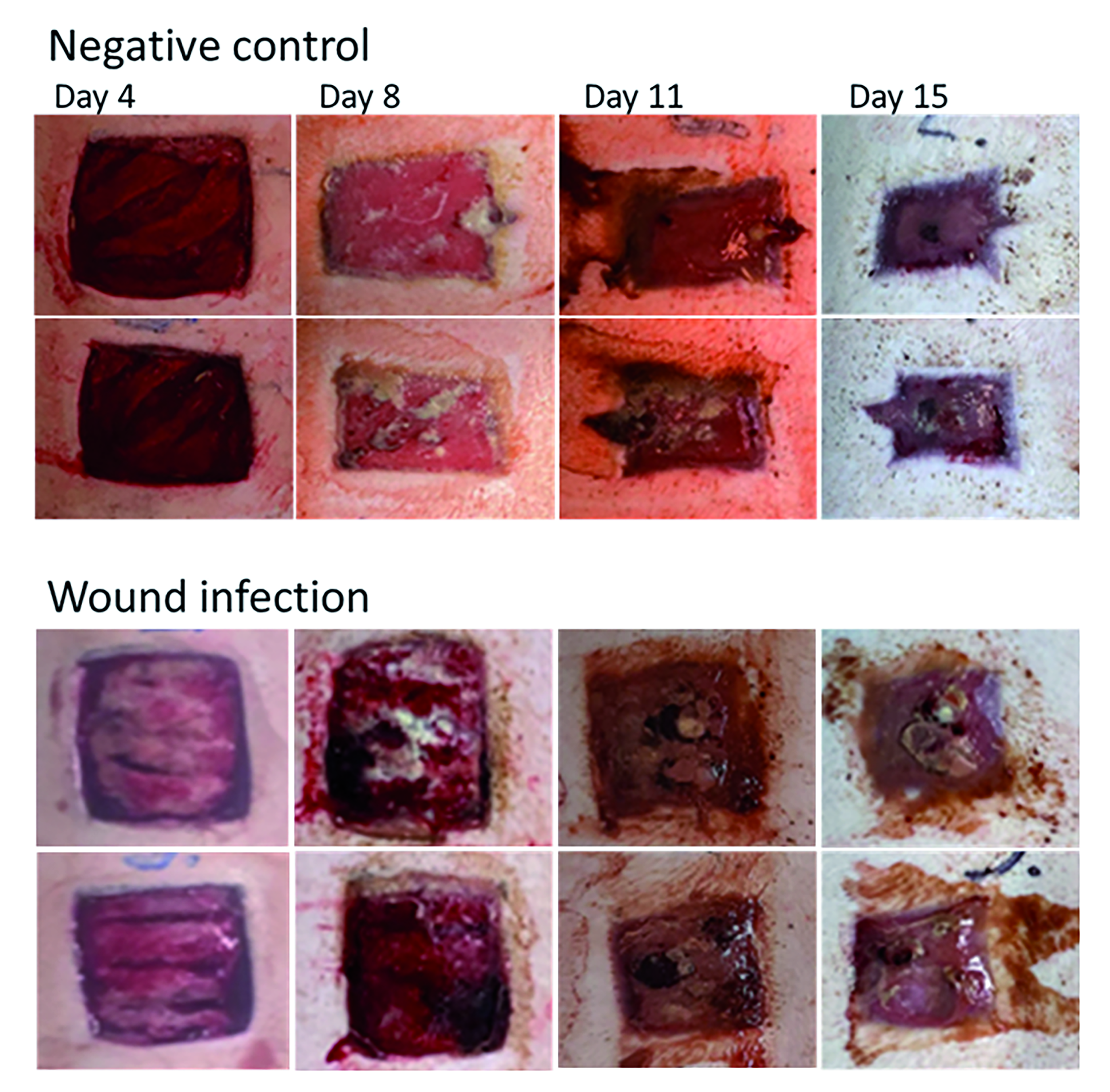

Supplement: Supplementary file 8 — Figure S8. [file AME2-8-544-s009.tif]
