# Supplementary material for: Development of a porcine model of skin and soft‐tissue infection caused by Staphylococcus aureus, including methicillin‐resistant strains suitable for testing topical antimicrobial agents
Source: Animal Model Exp Med. 2024 Oct 31;8(3):544–57. doi: 10.1002/ame2.12495 (PMC11904108; doi:10.1002/ame2.12495)
Supplement: Supplementary file 9 — Figure Captions. [file AME2-8-544-s008.docx]

# Development of a porcine model of skin and soft-tissue infection caused by *Staphylococcus aureus* including methicillin-resistant strains suitable for testing topical antimicrobial agents.

Filip Raška^1^, Břetislav Lipový^1,2^, Šárka Kobzová^3,4^, Lukáš Vacek^3,5^, Rea Jarošová^3,6^, Dominika Kleknerová^5^, Katarína Matiašková^3^, Peter Makovický^3,7^, Monika Vícenová^3^, Edita Jeklová^3^, Roman Pantůček^8^, Martin Faldyna^3^, Lubomír Janda^3*^

***Correspondence:** Lubomír Janda, lubomir.janda@vri.cz

# Supplementary Figures and Tables

**Supplementary Figure 1.** Fully repaired skin wound and on the left side massive granuloma (1) with completely superficial epidermal reepithelization (2) in comparison with normal skin on the right side. HE: 5x

**Supplementary Figure 2.** Tissue composing massive necrosis (1) and reparative activity with fibrotic areas, including groups of round-celullar inflammatory cells (2) and some remaining univacuolar fat cells (3). HE: 5x

**Supplementary Figure 3.** Part of the skin which is covered by continual epidermis with partially necrotic (1), partially fibrotic (2) oedematic (3) dermis. HE: 5x

**Supplementary Figure 4.** Almost totally fibrotic tissue with groups of round-cellular inflammatory cells (1), oedema (2) and superficially intensive superficial reepithelization (3). HE: 5x

**Supplementary Figure 5.** Tissue composing massive necrosis (1) and reparative activity with mass of erythrocytes (2) and some grups of round-cellular inflammatory cells (3). HE: 5x

**Supplementary Figure 6.** Tables for histopathological evaluation of samples.

**Supplementary Figure 7.** Table for clinical assessment of local signs of wound infection.

**Supplementary** **Figure 8**. Comparison of photographs of all skin and subcutaneous defects between wound infection and negative control on days 4, 8, 11 and 15.
